# High Frequency Sonoprocessing: A New Field of Cavitation‐Free Acoustic Materials Synthesis, Processing, and Manipulation

**DOI:** 10.1002/advs.202001983

**Published:** 2020-11-23

**Authors:** Amgad R. Rezk, Heba Ahmed, Shwathy Ramesan, Leslie Y. Yeo

**Affiliations:** ^1^ Micro/Nanophysics Research Laboratory School of Engineering RMIT University Melbourne VIC 3000 Australia

**Keywords:** crystallization, molecular self‐assembly, nanomaterials, polymers, sonochemistry, surface acoustic waves

## Abstract

Ultrasound constitutes a powerful means for materials processing. Similarly, a new field has emerged demonstrating the possibility for harnessing sound energy sources at considerably higher frequencies (10 MHz to 1 GHz) compared to conventional ultrasound (⩽3 MHz) for synthesizing and manipulating a variety of bulk, nanoscale, and biological materials. At these frequencies and the typical acoustic intensities employed, cavitation—which underpins most sonochemical or, more broadly, ultrasound‐mediated processes—is largely absent, suggesting that altogether fundamentally different mechanisms are at play. Examples include the crystallization of novel morphologies or highly oriented structures; exfoliation of 2D quantum dots and nanosheets; polymer nanoparticle synthesis and encapsulation; and the possibility for manipulating the bandgap of 2D semiconducting materials or the lipid structure that makes up the cell membrane, the latter resulting in the ability to enhance intracellular molecular uptake. These fascinating examples reveal how the highly nonlinear electromechanical coupling associated with such high‐frequency surface vibration gives rise to a variety of static and dynamic charge generation and transfer effects, in addition to molecular ordering, polarization, and assembly—remarkably, given the vast dimensional separation between the acoustic wavelength and characteristic molecular length scales, or between the MHz‐order excitation frequencies and typical THz‐order molecular vibration frequencies.

## Graphical abstract

Recent work has shown that high‐frequency acoustic vibration at MHz‐order frequencies offers vast opportunities for manipulating bulk/2D crystals and biological materials distinct from conventional sonochemical processes at lower frequencies, therefore opening up a new field of cavitation‐free materials synthesis and processing.

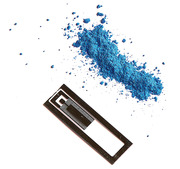

## 1 Introduction

### 1.1 Sonochemistry: Materials Synthesis Driven by Cavitation

**Figure 1:**
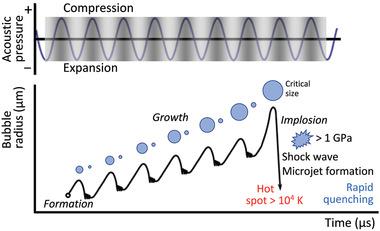
Schematic illustration of the transient acoustic cavitation process. Reproduced with permission.^[^ ^17^ ^]^ Copyright 2013, Royal Society of Chemistry.

Ultrasound has long been employed as a tool for synthesizing a wide range of materials. Where chemical reactions are involved, such ultrasound‐mediated synthesis has commonly been known by the term “sonochemistry.”^[^ ^1^, ^2^, ^3^, ^4^, ^5^ ^]^ Although convective mixing arising from the streaming (bulk liquid recirculation) generated when acoustic energy from ultrasonically vibrated structures is coupled into fluids (ref. [6] and references therein) has been reported for driving chemical synthesis (e.g., nanoparticle production through flash nanoprecipitation^[^ ^7^ ^]^), the predominant mechanism responsible for the majority of sonochemical synthesis can largely be attributed to acoustic cavitation.^[^ ^8^, ^9^, ^10^, ^11^ ^]^ In these events, a bubble or an ensemble of bubbles rapidly grows beyond a critical sound excitation intensity, oscillates and subsequently implodes (**Figure** **1**), thereby violently collapsing under the ultrasonic forcing to produce shock waves^[^ ^12^ ^]^ and high velocity (typically 100 m s^−1^) microjets.^[^ ^1^, ^13^ ^]^ The extreme pressures (>1 GPa) and temperatures (>10^4^ K) that result then act as a trigger for a number of phenomena. These include microstreaming, quenching, and free radical formation, which can be exploited to drive a number of physical processes, chemical reactions, or even biological effects over a wide range of scales, from localized microscopic hot spots in the vicinity of the collapsing bubble to bulk macroscopic acoustic streaming scales, commensurate with the length scales over which the acoustic energy dissipates.^[^ ^14^, ^15^, ^16^, ^17^, ^18^, ^19^ ^]^

The mechanical shear and turbulence associated with cavitation‐driven microstreaming, for example, has been employed for mixing,^[^ ^20^ ^]^ dispersion,^[^ ^21^ ^]^ emulsification,^[^ ^22^ ^]^ solvent extraction,^[^ ^23^ ^]^ and membrane filtration,^[^ ^24^ ^]^ in addition to enhancing mass transfer and reaction kinetics (i.e., sonocatalysis^[^ ^25^ ^]^). Rapid quenching effects arising from local temperature changes at the center of the collapsing bubble, on the other hand, have been reported to drive crystallization.^[^ ^26^ ^]^ Bond creation and cleaving under ultrasound are also useful for altering inter‐ or intra‐molecular interactions that can drive molecular self‐assembly such as lipid restructuring,^[^ ^27^ ^]^ sol–gel transitions, and other gelation processes.^[^ ^28^, ^29^, ^30^, ^31^ ^]^ Further, the generation of free radicals, arising from the dissociation of water molecules due to the breaking of OH bonds as a consequence of the intense local heating produced when cavitation bubbles collapse have been shown to be useful for initiating redox reactions in a variety of sonochemical processes. These include water splitting,^[^ ^32^ ^]^ metal ion reduction (e.g., in the synthesis of metal nanoparticles and metal/metal oxide composites for sensing, catalysis and optoelectronic applications^[^ ^33^, ^34^, ^35^, ^36^ ^]^), polymerization,^[^ ^37^ ^]^ electrodeposition,^[^ ^38^ ^]^ and the degradation of organic compounds.^[^ ^39^ ^]^

### 1.2 High‐Frequency Acoustic Excitation: The Possibility for Cavitation‐Free Materials Synthesis

**Figure 2:**
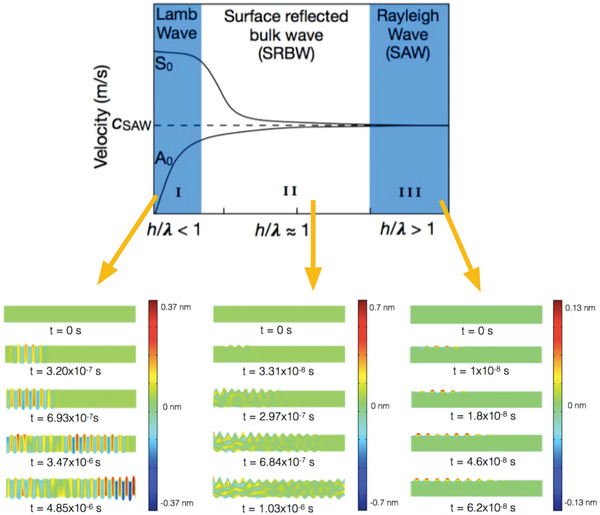
Bulk, surface and hybrid acoustic waves. Dispersion curves (top) and finite element simulations (bottom) showing the distinction between (I) a bulk (e.g., Lamb) wave, obtained for *h*/*λ* < 1; (II) a hybrid wave (i.e, a surface reflected bulk wave; SRBW), obtained for *h*/*λ* ≈ 1; and, (III) a Rayleigh surface wave (e.g., a surface acoustic wave; SAW), obtained for *h*/*λ* > 1. Here, the fundamental symmetric and asymmetric wave modes, denoted by *S*_0_ and *A*_0_, respectively, can be seen to asymptotically approach the SAW phase velocity *c*_SAW_ with decreasing wavelength *λ* (or increasing frequency) compared to the piezoelectric (in this case, lithium niobate (LiNbO_3_)) substrate thickness *h*. In the simulations, *t* denotes time. Reproduced with permission.^[^ ^40^ ^]^ Copyright 2016, Wiley‐VCH.

At frequencies beyond the conventional ultrasound range (16 kHz to 3 MHz), however, it is possible to generate a different nature of vibration to bulk ultrasonic excitation (*λ* ≫ *h* [regime I in **Figure** **2**]; see **Table** **1** for examples of these bulk waves that have been used for materials synthesis and processing) on or within a piezoelectric substrate of thickness *h*. This is because with higher frequencies, the wavelengths *λ* become progressively shorter and hence the sound energy becomes increasingly confined to the surface of the piezoelectric substrate.^[^ ^81^ ^]^ Consequently, the bulk acoustic wave is replaced by a surface acoustic wave (SAW) at sufficiently high frequencies when *λ* ≪ *h* (regime III in Figure 2).^[^ ^82^ ^]^ More recently, however, it was shown that a hybrid surface and bulk wave known as surface reflected bulk waves (SRBWs) can also exist in the intermediate transition regime where *λ* ≈ *h* (regime II in Figure 2).^[^ ^40^ ^]^

**Table 1:** Examples of the use of low‐frequency bulk acoustic waves for the synthesis and manipulation of various materials

Vibration mode	Wave description	Typical frequency range	Examples
Longitudinal	1D pressure wave (particle vibration displacement parallel to the direction of wave propagation) that is the dominant mode in horn transducers such as that employed in probe sonicators	20–100 kHz	Bulk crystals: Crystallization,^[^ ^41^, ^42^, ^43^, ^44^ ^]^ including that of metal–organic frameworks^[^ ^45^, ^46^ ^]^; crystal fragmentation^[^ ^47^ ^]^
			Nanomaterials: Liquid phase exfoliation of graphene,^[^ ^48^ ^]^ transition metal dichalcogenides,^[^ ^49^, ^50^ ^]^ and IV–VI binary semiconductors^[^ ^51^ ^]^ (including quantum dots and nanosheets); dispersion of carbon nanotubes^[^ ^52^ ^]^
			Polymeric and biological materials: Emulsion‐based polymerization^[^ ^53^ ^]^; polymer nanoparticle synthesis^[^ ^54^ ^]^
Plate (thickness expansion mode)	Vibration occurs in the direction of the plate thickness, which determines the resonant frequency; dominant mode associated with the transducers used in bath sonicators and ultrasonic nebulizers (e.g., for spray pyrolysis)	1 kHz–1 MHz, although higher‐frequency operation (up to GHz) can be achieved with ultrathin plate resonators	Ultrasonic bath (typically 1–50 kHz)
			Bulk crystals: Crystallization,^[^ ^55^ ^]^ including that of metal–organic frameworks^[^ ^56^, ^57^ ^]^
			Nanomaterials: Liquid phase exfoliation of graphene^[^ ^58^ ^]^ and transition metal dichalcogenides^[^ ^59^, ^60^ ^]^; synthesis of carbon nanotubes,^[^ ^61^ ^]^ nanofibers,^[^ ^62^ ^]^ and liquid metal droplets^[^ ^63^ ^]^
			Polymeric and biological materials: Polymer synthesis^[^ ^64^, ^65^ ^]^; transdermal drug delivery^[^ ^66^ ^]^; intracellular delivery^[^ ^67^, ^68^, ^69^ ^]^
			Ultrasonic nebulizers (typically 1 kHz–2 MHz)
			Bulk crystals: Spray pyrolysis for nanoparticle and nanowire synthesis^[^ ^70^, ^71^, ^72^, ^73^, ^74^ ^]^
			Nanomaterials: Spray pyrolysis for nanocrystallization of molten salts,^[^ ^75^ ^]^ synthesis of porous transitional metal dichalcogenides and their composites,^[^ ^76^, ^77^ ^]^ and, thin film deposition^[^ ^78^ ^]^
			Polymeric and biological materials: pulmonary drug delivery of aerosols and nanoparticles^[^ ^79^, ^80^ ^]^

Moreover, cavitation becomes increasingly difficult to induce and, in our experience, is essentially non‐existent in SAW or SRBW excitation. This is not only due to the increase in the threshold sound intensity^[^ ^83^, ^84^, ^85^, ^86^ ^]^ needed to trigger these events with increasing frequency,^[^ ^34^, ^87^, ^88^ ^]^ but also because the greater efficiencies of these systems often necessitate far lower input energies to drive similar mechanical phenomena (e.g., acoustic streaming in liquids) compared to their low‐frequency counterparts.^[^ ^89^, ^90^, ^91^, ^92^ ^]^

In spite of this, these high‐frequency surface and hybrid waves have been observed to drive similar phenomena, albeit via completely different physicochemical mechanisms that do not involve cavitation.^[^ ^93^ ^]^ Indeed, the possibility for cavitation‐free sonochemistry was conjectured in a recent opinion paper penned by the former Editor‐in‐Chief of *Ultrasonics Sonochemistry* Prof. Timothy Mason.^[^ ^94^ ^]^ A recent example is the increase in catalytic oxidation activity upon exposure of platinum catalysts to the SAW, which was attributed to the role of picosecond order localized fluctuations in the form of shock wave pulses that the SAW generates during each cycle. The shock wave propagation through the atomic lattice of the catalyst was then suggested to lead to a dynamic enhancement in the diffusion, desorption, and reaction rates.^[^ ^95^ ^]^

Another recent example is the possibility of generating free radicals with the SAW that leads to the dissociation of pure water or the decomposition of dyes in the absence of catalysts, electrolytes, or even electrode contact.^[^ ^93^ ^]^ Unlike cavitation‐driven sonolytic water splitting,^[^ ^32^ ^]^ the dissociation of the water molecules was shown to be a consequence of the strong electromechanical coupling associated with the propagation of the acoustic wave on the piezoelectric substrate, whose evanescent electric field will be seen subsequently to be responsible for a host of other phenomena related to materials synthesis, processing, and manipulation. In this case, the coupled electromechanical effects of both the physical undulation of the mechanical wave together with the evanescent electric field produces a high field intensity (>10^7^ V m^−1^) sufficient to drive self‐ionization of water. The large field strength is a result of the highly confined polarized regions that arise when liquid is trapped within the nanometer‐order amplitude of the acoustic wave potential; the enhanced polarization being a consequence of this confinement length scale, which is much smaller than the Debye length (around 300 nm for deionized water). Moreover, the free radical generation, and subsequent dye decomposition, appeared to be suppressed when the electric field component of the SAW was screened and hence decoupled from the mechanical wave. Such an observation thus further supports the role of the electromechanical coupling over a cavitation‐dominant mechanism since free radical generation by a pure mechanical wave via bulk ultrasonic excitation has been observed in cavitation‐induced sonolysis.

**Figure 3:**
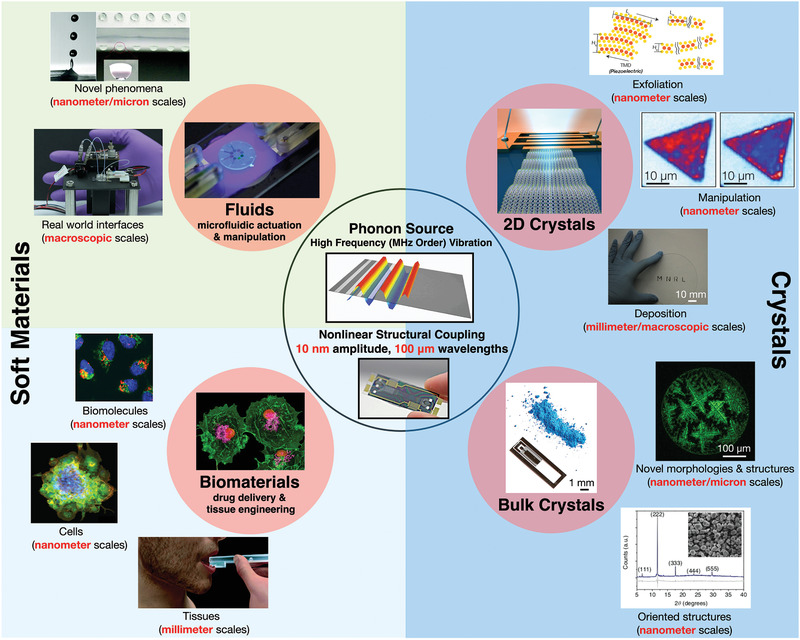
Snapshot of the novel nonlinear multiscale phenomena and potential applications arising from fluid– and material–structure interactions associated with the coupling of phonon energy from MHz‐order acoustic forcing into either fluids or bulk/2D crystalline and biological materials. The highlighted text in red indicates the many different length scales over which the coupling remarkably occurs. While various hypotheses have been discussed, a definitive and unifying framework for the underlying physicochemical mechanism responsible for the coupling is still an open scientific question (Novel phenomena: Reproduced with permission.^[^ ^161^ ^]^ Copyright 2018, Royal Society of Chemistry. Real world interfaces: Reproduced with permission.^[^ ^200^ ^]^ Copyright 2014, American Chemical Society. Cells: Reproduced with permission.^[^ ^148^ ^]^ Copyright 2016, American Chemical Society. Tissues: Reproduced with permission.^[^ ^146^ ^]^ Copyright 2018, Royal Society of Chemistry. Oriented structures: Reproduced under a CC‐BY 4.0 license.^[^ ^115^ ^]^ Published by Springer Nature. Deposition; Exfoliation: Reproduced with permission.^[^ ^123^ ^]^ Copyright 2018, Wiley‐VCH. Manipulation: Reproduced with permission.^[^ ^131^ ^]^ Copyright 2016, American Chemical Society).

In a similar manner to the electromechanical water splitting example above, we shall discuss below the amenability of these high‐frequency surface and hybrid acoustic waves (i.e., the SAW and SRBW) as a vehicle to drive the synthesis, manipulation, and processing of various 2D and bulk crystalline as well as polymeric and other materials (**Table** **2**), therefore suggesting the dominant role of fundamental physicochemical mechanisms other than cavitation in these processes. In particular, we highlight recent studies that report these observations, which likely arise as a consequence of the nonlinear high‐frequency material–structure interactions (such as the electromechanical coupling described above), in much the same way high‐frequency fluid–structure interactions have in the past decade been shown to drive a number of unique phenomena, including thin film fingering instabilities, solitary wave dynamics, and capillary wave turbulence, among others^[^ ^96^, ^97^, ^98^, ^99^ ^]^ (**Figure** **3**).

**Table 2:** Summary of work to date on high‐frequency acoustic synthesis and manipulation of various materials

**Bulk crystals**
*Alignment, patterning, and manipulation*
Acoustic radiation force
‐ Particle/bioparticle trapping at nodes/antinodes for in situ polarization, gelation, and crystallization^[^ ^100^, ^101^, ^102^, ^103^ ^]^
Acoustic streaming
‐ Molecular reorientation of liquid crystals^[^ ^104^ ^]^
‐ Particle/bioparticle aggregation and concentration^[^ ^105^, ^106^, ^107^, ^108^, ^109^, ^110^, ^111^ ^]^
*Synthesis*
Microcentrifugation
‐ Enhancement of chemical/biochemical reactions^[^ ^112^, ^113^, ^114^ ^]^
‐ Crystallization of metal–organic frameworks^[^ ^115^ ^]^
Nebulization
‐ Crystallization of inorganic salts, organic compounds, and metal–organic frameworks^[^ ^116^, ^117^, ^118^ ^]^
**Nanomaterials**
*Alignment and patterning*
‐ Electric field and acoustic radiation force assisted nanoparticle, nanocolloid, nanowire, nanotube assembly^[^ ^119^, ^120^, ^121^, ^122^ ^]^
*Exfoliation*
‐ Nebulization of mono‐ and few‐layer quantum dots and nanosheets^[^ ^123^, ^124^ ^]^
‐ Microcentrifugation of mono‐ and few‐layer nanosheets^[^ ^125^ ^]^
‐ Mechanical (dry) synthesis of quantum dots and large flakes^[^ ^126^ ^]^
*Manipulation of electronic band structures*
‐ Thin semiconductor films in the presence of 2D electron gases^[^ ^127^, ^128^, ^129^ ^]^
‐ Multi‐, few‐, and mono‐layer transition metal dichalcogenides^[^ ^130^, ^131^ ^]^
‐ Heterojunction p‐n diodes^[^ ^132^ ^]^
**Polymeric and biological materials**
*Cell/organism/tissue manipulation*
‐ Facilitation of cell–cell interactions^[^ ^133^ ^]^
‐ Piezochannel activation^[^ ^134^, ^135^ ^]^
‐ Stimulation of exosome production^[^ ^136^ ^]^
‐ Acoustotaxis^[^ ^137^ ^]^
‐ Tissue oxygenation and wound healing^[^ ^138^ ^]^
‐ Nematode stimulation^[^ ^139^, ^140^ ^]^
‐ Lipid structure reorganization^[^ ^141^, ^142^ ^]^
‐ Intracellular uptake^[^ ^142^ ^]^
‐ Cell lysis^[^ ^143^, ^144^, ^145^ ^]^
‐ Tissue permeabilization and delivery^[^ ^146^ ^]^
*Cell culture*
‐ Enhancing flow perfusion in culture systems^[^ ^147^ ^]^
‐ Spheroid production^[^ ^148^, ^149^, ^150^ ^]^
*Polymer nanoparticle production*
‐ Nebulization‐driven synthesis and encapsulation of nanoparticles and multilayer capsules^[^ ^151^, ^152^, ^153^, ^154^, ^155^ ^]^
*Patterning and deposition*
Nebulization
‐ Spray coating of functional thin films^[^ ^156^ ^]^
‐ Protein microarray spot patterning^[^ ^157^ ^]^
Acoustowetting
‐ Polymer/hydrogel films^[^ ^158^, ^159^, ^160^ ^]^
Jetting
‐ Single cell deposition^[^ ^161^ ^]^
‐ Sample dispensing and patterning^[^ ^161^, ^162^, ^163^ ^]^
*Inhalation drug delivery and mass spectrometry interfacing*
‐ Nebulization of biologics^[^ ^154^, ^164^, ^165^, ^166^, ^167^, ^168^, ^169^, ^170^, ^171^ ^]^

## 2 Bulk Crystals

### 2.1 Alignment, Patterning, and Manipulation

**Figure 4:**
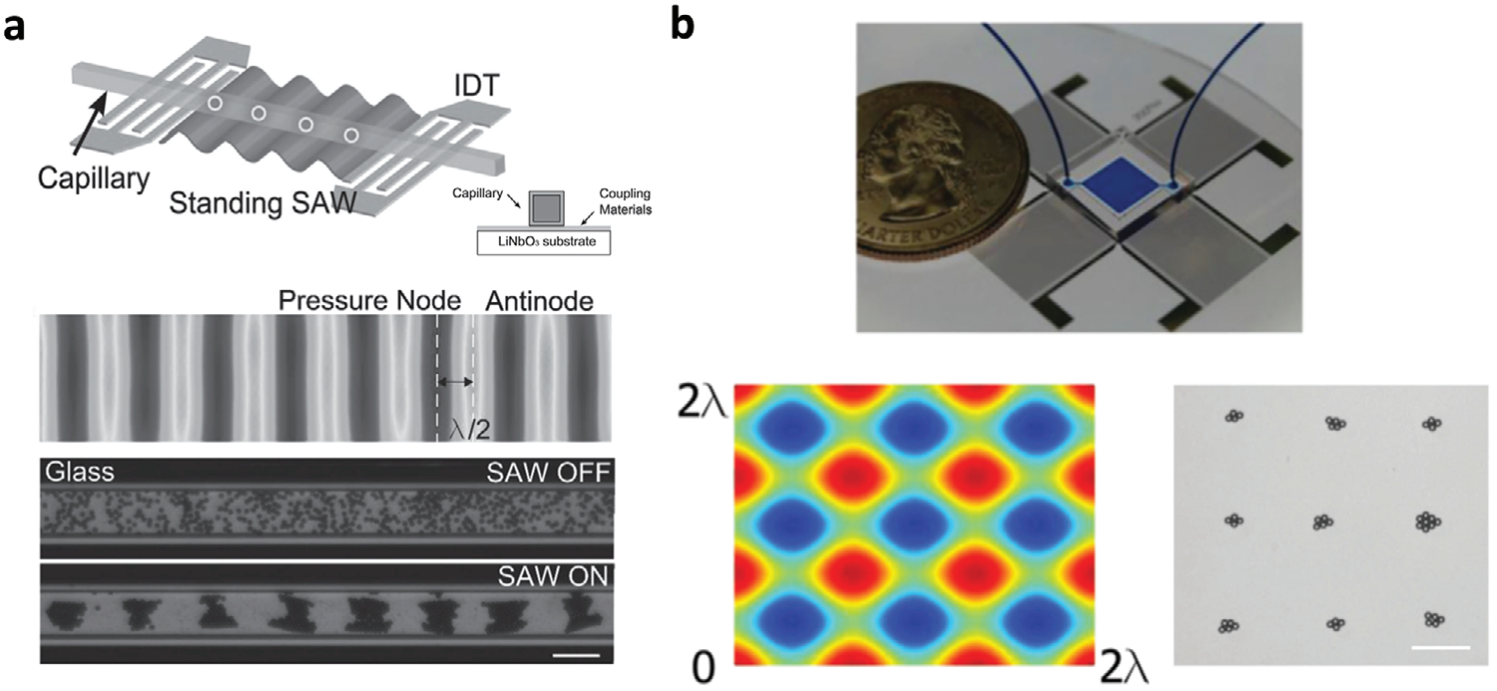
Patterning of protein crystals in a capillary tube along nodal positions of a standing SAW. a) The top pair of images show schematic illustrations of the experimental setup in which a standing SAW—generated by applying an AC electrical signal at resonance to interdigitated transducer (IDT) electrodes on the piezoelectric LiNbO_3_ substrate—is transmitted through a couplant into a square capillary (see inset image) to set up a standing bulk wave within it. The bottom pair of images show the aggregation of protein crystals in an originally dispersed suspension within the capillary onto lines associated with the pressure nodes, which are separated by half sound wavelengths *λ*/2, upon application of the SAW. b) Instead of linear protein crystal aggregates, it is also possible to form a 2D array of protein crystal aggregate spots (bottom right image) precisely atop the nodes associated with the standing SAW pattern on the device (bottom left image) through the addition of a transverse IDT pair (top image). The scale bars denote length scales of 100 µm. Reproduced with permission.^[^ ^178^ ^]^ Copyright 2015, Wiley‐VCH.

At sufficiently low acoustic intensities where the flow is quiescent, particles or cells suspended in a liquid exposed to a standing sound wave can be transported under the acoustic radiation force (at times, in conjunction with other forces, e.g., dielectrophoretic^[^ ^172^, ^173^ ^]^ or magnetic^[^ ^174^ ^]^) and trapped at either its pressure nodes or antinodes along the substrate surface, depending on their acoustic contrast factor.^[^ ^175^, ^176^ ^]^ This simple concept has been used extensively in microfluidic devices,^[^ ^91^, ^92^, ^177^ ^]^ for example, to localize cross‐linking at these predefined positions along nodal positions so as to enable in situ polarization^[^ ^100^ ^]^ or gelation.^[^ ^101^, ^102^ ^]^ Similarly, it is also possible to pattern protein crystals along nodal lines which are separated by a distance of half sound wavelengths, induced in the fluid comprising a protein suspension that is housed in a capillary atop a SAW device, as illustrated in the schematic in **Figure** **4**a.^[^ ^178^ ^]^ We note that although a standing SAW is produced on the piezoelectric substrate, its transmission through a thin coupling layer that is sandwiched between the SAW substrate (in this case, lithium niobate (LiNbO_3_)) and the capillary sets up a standing bulk sound wave in the capillary.^[^ ^91^, ^179^ ^]^ 2D spot patterns of these crystals can also be obtained through the inclusion of an orthogonal electrode pair to set up a corresponding standing SAW in the transverse direction, as depicted in Figure 4b. The size of the crystal patterns that can be formed is nevertheless limited upon saturation of the pressure nodes (or antinodes). To circumvent this, travelling SAWs were instead proposed, for example, to generate a counteracting acoustic radiation force on colloidal particles flowing in a microchannel to oppose the hydrodynamic drag imposed on them by the flow, so as to effect their stagnation and hence packing into a colloidal crystal within the channel.^[^ ^103^ ^]^

**Figure 5:**
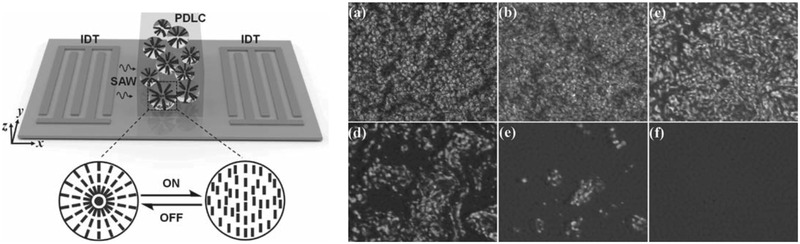
Manipulation of polymer‐dispersed liquid crystals under SAW excitation. The left panel shows a schematic depiction of the experimental setup in which a travelling SAW, generated on the piezoelectric substrate by applying an AC electrical signal at the resonant frequency of the interdigitated transducer (IDT) electrodes, is transmitted into a film consisting of a cured polymer‐dispersed liquid crystal (PDLC) matrix that is placed atop the substrate. The right panel illustrates the effect of 34 dBm power of SAW on the liquid crystal morphology, a) initially, prior to the SAW excitation, and after b) 20 s, c) 40 s, d) 60 s, e) 80 s, and f) 100 s. Reproduced with permission.^[^ ^104^ ^]^ Copyright 2011, Wiley‐VCH.

On the other hand, the diffraction of the SAW (or SRBW) and hence the leakage of its energy into the liquid phase in contact with the piezoelectric substrate along which the acoustic wave propagates—a consequence of the mismatch in sound speeds between the solid and liquid phases—gives rise to the propagation of bulk sound waves into the liquid, whose attenuation over a length scale

(1) $$\begin{array}{c} \beta^{-1}=\frac{\omega^{2}}{\rho c^{3}}\left(\frac{4\mu }{3}+\mu_{B}\right) \end{array}$$

gives rise to bulk vortical flow in the liquid known as acoustic streaming.^[^ ^180^, ^181^, ^182^, ^183^ ^]^ In the above, *ω* is the acoustic frequency, *ρ* the density of the liquid, *c* its corresponding sound speed, and, *μ* and *μ*_B_ its shear and bulk viscosity, respectively. Over the past decade, this nonlinear coupling of energy from the solid phase into the liquid has provided a means for driving efficient microfluidic actuation for a wide range of applications,^[^ ^89^, ^90^, ^91^, ^92^ ^]^ for example, drop transport^[^ ^184^, ^185^, ^186^, ^187^, ^188^, ^189^ ^]^ and manipulation,^[^ ^190^ ^]^ in addition to microchannel/nanochannel actuation.^[^ ^191^, ^192^, ^193^, ^194^, ^195^, ^196^, ^197^, ^198^, ^199^ ^]^ The SAW‐driven acoustic streaming was also demonstrated as a means to manipulate nematic liquid crystals in a polymer film atop the SAW substrate by facilitating molecular re‐orientation under the flow, aided by the decrease in viscosity and birefringence of the crystals as a consequence of the temperature increase due to absorption of the sound waves in the liquid.^[^ ^104^ ^]^ More specifically, the flow recirculation was observed to re‐orientate the crystals in a direction orthogonal to that of the SAW propagation, as shown in **Figure** **5**, thus enabling the possibility of reversible switching and hence an acousto‐optic shuttering effect.

### 2.2 Synthesis

#### 2.2.1 Microcentrifugation‐Driven Crystallization

**Figure 6:**
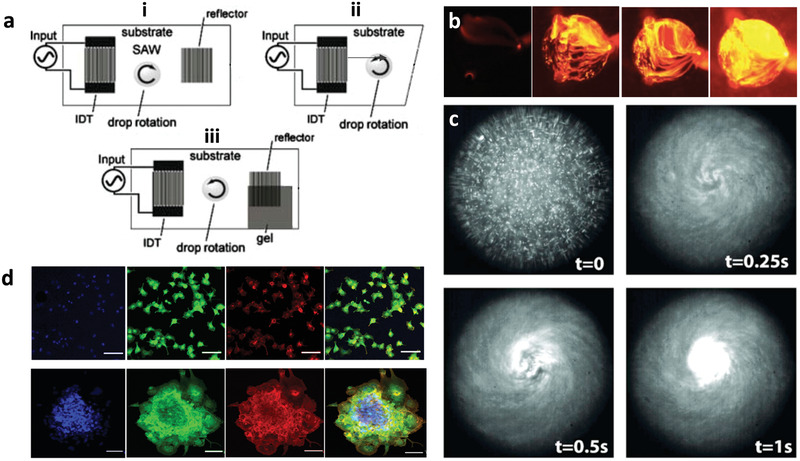
SAW microcentrifugation flow. a) Azimuthal rotational streaming flow in a sessile drop atop the piezoelectric substrate can be generated by breaking the symmetry of the SAW irradiation into the drop, either by i) offsetting the position of the drop such that only part of it lies along the SAW irradiation pathway, ii) making an asymmetric cut to the substrate such that the reflection of the SAW is laterally nonuniform with respect to the drop, or iii) absorbing part of the SAW such that it is prevented from being reflected back into the drop. b) Top view images showing chaotic mixing in the drop (or chamber; ≈1 mm in diameter) driven by the SAW microcentrifugation flow; the image sequence shows the effect of increasing the SAW power with the first image being the control (i.e., no SAW excitation). c) Rapid concentration of 500 nm fluorescent particles in the drop driven by the SAW microcentrifugation flow. d) Formation of 3D cell spheroids by SAW‐driven cell aggregation (bottom row) compared to the 2D monolayers that form in the absence of the SAW microcentrifugation flow (top row) (first column: 4′,6‐diamidino‐2‐phenylindole (DAPI) nuclear staining, second column: vinculin focal contact staining, third column: phalloidin F‐actin staining, fourth column: overlay; the scale bar denotes 100 µm lengths). a) Reproduced with permission.^[^ ^105^ ^]^ Copyright 2007, Springer Science + Business Media LLC. b) Reproduced with permission.^[^ ^200^ ^]^ Copyright 2014, American Chemical Society. c) Reproduced with permission.^[^ ^106^ ^]^ Copyright 2008, American Institute of Physics. d) Reproduced with permission.^[^ ^148^ ^]^ Copyright 2016, American Chemical Society.

If the SAW irradiation into the liquid atop the SAW substrate is transversely (orthogonal to the SAW propagation direction) distributed (examples of how symmetry breaking of the SAW can be generated by various means is shown in **Figure** **6**a), an azimuthal microcentrifugal acoustic streaming flow within the liquid^[^ ^105^, ^106^ ^]^ arises. Such a flow potentially constitutes a convective energy source that could enhance the rate and yield of chemical and biochemical reactions,^[^ ^112^, ^113^, ^114^ ^]^ possibly by overcoming diffusional transport limitations as well as activation energy barriers—the latter through the picosecond shocks that the SAW produces,^[^ ^95^ ^]^ as alluded to above. Additionally, the SAW microcentrifugation can be utilized for driving efficient microfluidic mixing, sample enrichment, and particle separation (see, e.g., Figure 6b,c).^[^ ^105^, ^106^, ^108^, ^200^ ^]^ The ability to rapidly and efficiently concentrate particles to a focal spot^[^ ^105^, ^106^, ^107^, ^108^, ^109^, ^110^ ^]^ also allowed for the aggregation of colloidal particles from a bulk suspension that subsequently assembled into a crystalline structure^[^ ^111^ ^]^ akin to a similar process that used bulk ultrasound to enhance colloidal self‐assembly.^[^ ^201^ ^]^

**Figure 7:**
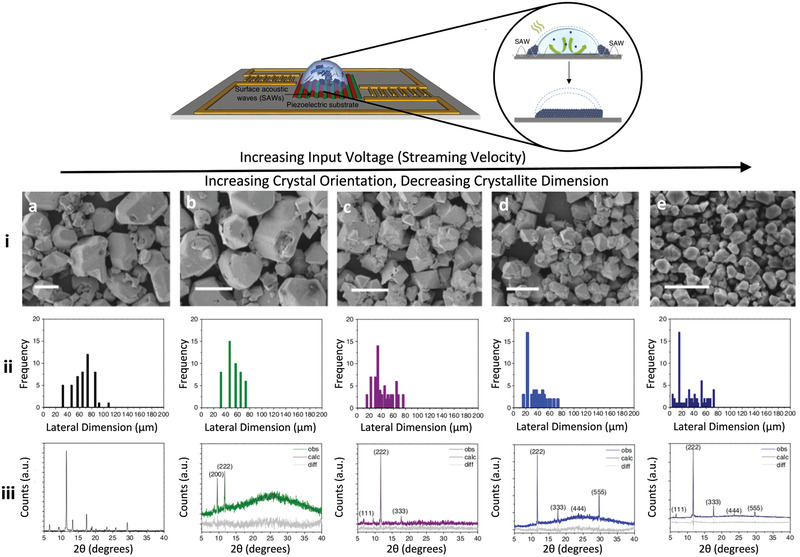
SAW microcentrifugation synthesis of MOFs. The top shows the experimental setup in which two opposing asymmetric SAWs, generated by offset interdigitated transducers at each end, drive an azimuthal microcentrifugation flow within a sessile drop consisting of the MOF precursor solution comprising 1,3,5‐benzenetricarboxylic acid (BTC; C_6_H_3_(CO_2_H)_3_) and copper(II) nitrate hemi(pentahydrate) (Cu(NO_3_)_2_ · 2.5 H_2_O). As illustrated in the inset, the MOF powder that crystallizes on the substrate after 5 min of SAW excitation appears to possess out‐of‐plane (in the direction transverse to the substrate) orientation. This is evident in the experimental results below, which show that the crystals not only progressively decrease in size from i) the atomic force microscopy (AFM) images and ii) the particle size distributions, but are also increasingly oriented from iii) the x‐ray diffraction (XRD) spectra as the input voltage and hence the SAW power is increased from a) 0 V (i.e., control experiment without SAW excitation), b) 1.5 V, c) 4.5 V, d) 7.5 V, and e) 9 V. Scale bars denote a length scale of 50 µm. Also shown in the XRD results are the Pawley fits (calc) to the experimental (obs) data, as well as the difference between them (diff). Adapted with permission under a CC‐BY 4.0 license.^[^ ^115^ ^]^ Published by Springer Nature.

Crystallization of metal–organic frameworks (MOFs)—highly ordered 3D coordination networks comprising inorganic nodal units interconnected by polytopic organic ligands—under such microcentrifugation flows has also been reported. In this scenario, the enhanced convective solutal transport by the flow to the receding evaporating region at the contact line of the drop enriches the local solutal concentration (**Figure** **7**) in a manner which, in combination with the molecular dipole polarization under the evanescent electric field that accompanies the SAW due to the piezoelectric effect, promotes long‐range 3D superlattice ordering of vertically stacked monolayers.^[^ ^115^ ^]^ This results in preferential out‐of‐plane orientation of the MOFs that are rapidly (over several minutes) synthesized under these conditions, as observed by the x‐ray diffraction (XRD) spectra in Figure 7b–e. The degree of orientation is seen to increase with the SAW irradiation to the drop and hence the intensity of the convective flow, in stark contrast to the random orientation obtained in the absence of the SAW excitation under the diffusion‐dominant solutal transport within the slowly evaporating drop (Figure 7a).

That such acoustic excitation is able to directly influence molecular ordering to produce these highly oriented MOFs is quite unexpected. This is due to the long‐held belief that high‐frequency acoustic excitation >1 MHz would not influence the excited medium, particularly in the context of crystallization.^[^ ^202^ ^]^ Moreover, there is a stark discrepancy between the molecular length scales and the sound wavelength (typically 100 µm order), such that direct interaction between the sound waves with the molecules at these scales is not expected.^[^ ^35^ ^]^ We note though similar precedents in which bulk ultrasound has been reported to facilitate activation of surface processes such as surface diffusion of atomic clusters and their ejection via desorption from the surface. A similar mismatch is present in the latter, albeit between the excitation frequency and the THz‐order frequency associated with the vibrational states of the atomic clusters. There, a different nonlinear mechanism was proposed, involving the sharpening of the SAW wavefront into a shock wave. This, in turn, gives rise to higher‐frequency harmonics that directly couples the acoustic energy dynamically to thermal phonons associated with the vibrational modes of the atomic clusters,^[^ ^203^ ^]^ therefore activating surface diffusion processes that can eject or desorb the atomic clusters from the surface.^[^ ^204^ ^]^ We note, quite interestingly though, that similar orientation was not obtained in MOFs synthesized sonochemically through conventional bulk ultrasound,^[^ ^205^, ^206^, ^207^ ^]^ neither were these observed to be simultaneously activated compared to the MOFs produced by the SAW microcentrifugation.^[^ ^115^ ^]^

#### 2.2.2 Nebulization‐Driven Crystallization

**Figure 8:**
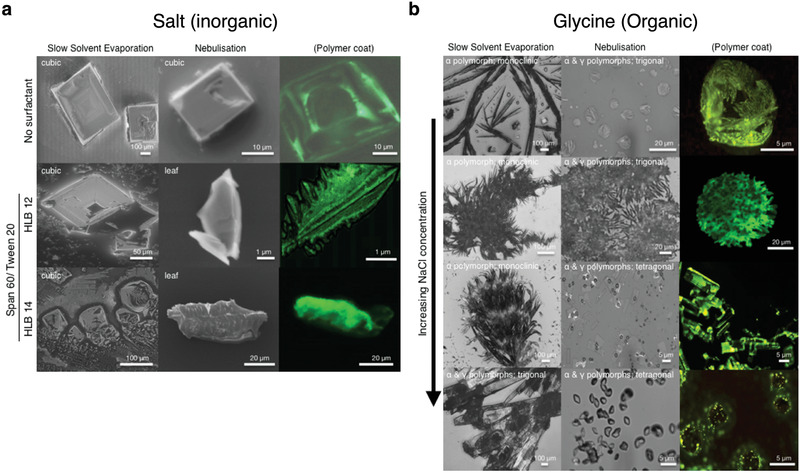
Novel crystal morphologies obtained via SAW/SRBW nebulization of a) salt and b) glycine solutions. The left columns in both panels show the crystals obtained under slow solvent evaporation whereas the middle and right columns show that obtained under the SAW/SRBW nebulization. The right column depicts the polymer‐coated crystals obtained when the solution that is nebulized additionally contains a fluorescent polymer. The results for the salt crystals are shown for surfactant solutions with different hydrophilic‐lipophilic balances (HLB) of the surfactant mixture (Span 60 and Tween 20) and those for glycine are shown for increasing sodium chloride (NaCl) concentration. Reproduced with permission.^[^ ^116^ ^]^ Copyright 2017, Wiley‐VCH.

The extremely large surface acceleration ($\approx \;O(10^{8}$ m s^−2^)) along the piezoelectric substrate as the SAW or SRBW propagates along or through it, on the other hand, constitutes an efficient means for generating aerosols in the 1–10 µm diameter range through a nebulization process,^[^ ^208^, ^209^, ^210^, ^211^, ^212^ ^]^ that has, to date, been demonstrated as a potent platform for portable inhaled therapeutics,^[^ ^164^ ^]^ and as an ionization source for mass spectrometry.^[^ ^154^, ^169^, ^170^, ^171^ ^]^ In a manner akin to spray‐drying techniques, nebulization of NaCl solutions with the hybrid acoustic waves was shown to give rise to the formation of crystals.^[^ ^116^ ^]^ Unlike spray drying, and distinct from ultrasonic annealing,^[^ ^213^ ^]^ however, the nebulization process was observed to produce crystals with uniquely new morphologies with orthorhombic packing besides the typical cubic structures reported to date for NaCl^[^ ^116^ ^]^ (**Figure** **8**). This was attributed to the unique intermediate evaporation rate regime (≈10^−5^–10^−6^ l h^−1^) associated with the drying of the aerosols containing the crystal stock solution in‐flight in air following their nebulization from the liquid surface. This evaporation rate regime straddles the extremely fast evaporation rates that accompany typical spray drying processes in which the aerosols in‐flight traverse through a sheath‐air flow heated to elevated temperatures (≈10^−1^–10^0^ l h^−1^), and the slow evaporation rates associated with slow solvent evaporation (≈10^−11^–10^−12^ l h^−1^).

The different crystal morphologies were not only limited to inorganic salts. When an organic solution (glycine) was nebulized using the same hybrid acoustic wave platform, a different polymorph (*γ*), known to form only under considerably more laborious conditions, was observed with different lattice packing compared to the typical polymorph (*α*) obtained under slow solvent evaporation (Figure 8); the relative ratios in the *α* and *γ* polymorphs depending on the NaCl concentration added. SAW nebulization was also recently employed in the generation of amorphous nanoparticles, although we note the role of the acoustics in that case was limited to the generation of aerosols and a downstream drying section involving the usual heated sheath compressed air flow was used. In this regard, the process is thus more akin to spray drying.^[^ ^117^ ^]^

**Figure 9:**
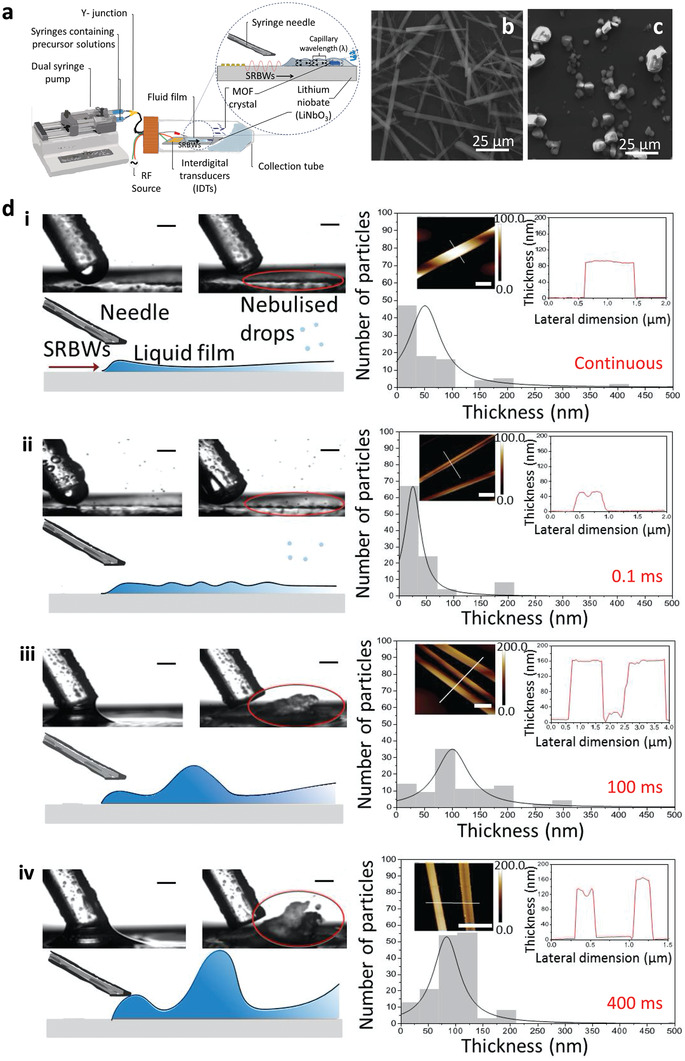
Synthesis of large aspect ratio MOF crystals with exposed active metal sites from thin nebulizing acoustowetting films. a) Experimental setup in which the MOF precursor solution comprising 1,3,5‐benzenetricarboxylic acid (BTC; C_6_H_3_(CO_2_H)_3_) and copper(II) nitrate hemi(pentahydrate) (Cu(NO_3_)_2_ · 2.5 H_2_O) is dispensed using a dual syringe pump onto the substrate, which then spreads under the SRBW into a thin film that concurrently nebulizes. The Cu–BTC MOF crystals that precipitate out in the thin liquid film possess b) a 1D sword‐like morphology and unique P2_1_/n monoclinic structure compared to c) the regular Fm3m cubic HKUST‐1 crystals that are usually obtained through the conventional bulk solvothermal technique (i.e., the control experiment in the absence of the SRBW forcing). Moreover, the thicknesses of the sword‐like crystals correspond to the liquid film thickness (as illustrated by the red circles), which, in turn, correlates strongly with d) the pulse duration (50% duty cycle) associated with the modulation of the acoustic excitation signal: i) continuous excitation (i.e., infinitesimally small pulse duration), ii) 0.1 ms, iii) 100 ms, iv) 400 ms. The scale bars in the monographs in the left column correspond to a length of 500 µm, whereas those in the atomic force microsopy (AFM) images in the insets of the right column correspond to a length of 1 µm. Reproduced with permission.^[^ ^118^ ^]^ Copyright 2020, Royal Society of Chemistry.

On the other hand, the nebulization of acoustowetting films^[^ ^96^, ^98^ ^]^ were also observed to produce distinct 1D crystal morphologies.^[^ ^118^ ^]^ In this case, a MOF precursor solution similar to that used previously in ref. [115] was drawn by the SRBW from a reservoir through a nozzle tip onto the surface of the device where it spread into a thin acoustowetting film,^[^ ^96^, ^98^ ^]^ while being simultaneously nebulized, under the SRBW forcing (**Figure** **9**a) to produce 1D sword‐like crystal morphologies with corresponding P2_1_/n monoclinic space groups (Figure 9b). These were quite distinct from the 3D cubic Fm3m structures usually obtained through conventional bulk solvothermal synthesis starting from the same precursor solutions (Figure 9c). While the lengths of these 1D sword‐like crystals are a result of the lateral constriction imposed by the 100 µm order wavelengths of the resonant capillary waves on the free surface of the thin film that are harmonically induced by the SRBW on the underlying substrate,^[^ ^97^, ^214^ ^]^ their thicknesses (10–100 nm) are strongly correlated with the film height, which, in turn, can be tuned through the pulse duration associated with modulation of the acoustic excitation signal (Figure 9d). We note an optimum exists (0.1 ms pulse duration; Figure 9d‐ii) where the film height and hence the sword‐like crystals that are produced are at their thinnest, due to the balance between the rate at which the liquid is drawn onto the substrate by the SRBW with the rate at which it leaves the substrate via nebulization such that the film residence time is minimized. In addition, the small crystal thicknesses are also a consequence of the rapid solvent evaporation associated with the nebulization off the thin film, which arrests further crystal growth in the vertical direction. More interestingly, we found the metal (in this case, copper) nodes, which are usually buried internally within the 3D coordination network structure of the MOF, to be exposed on the 1D crystal surface by virtue of their thicknesses. Besides enhancing the electrical conductivity of the MOF, such exposure of the metal at the surface of the crystal facilitates greater accessibility to these active sites to enhance catalytic binding activity, therefore rendering this novel synthesis procedure a rapid and facile alternative to the complex pre‐ or post‐synthesis chemical modification strategies currently employed for metal site exposure.^[^ ^118^ ^]^

The nebulization setup further offers the opportunity for continuous production of MOFs, constituting a significant advantage over the conventional batch processes that are commonly used, which, despite their ability for MOF production on kg scales, suffer from limited space–time yields (i.e., low production rates).^[^ ^215^ ^]^ The production rate of 4 g h^−1^ per device, which is equivalent to a theoretical space–time yield of approximately 36 000 kg m^3^ day^−1^,^[^ ^118^ ^]^ is significantly higher than the typical 10^3^ kg m^3^ day^−1^ values reported with bulk ultrasound MOF processing.^[^ ^216^ ^]^ Moreover, given the low cost and small footprint of the chipscale device, the throughput can be further increased by utilizing multiple devices in parallel to obtain space–time yields comparable to or even exceeding that reported with a microreactor flow‐through setup of up to 210 000 kg m^−3^ day^−1^.^[^ ^217^ ^]^

## 3 Nanomaterials

### 3.1 Alignment and Patterning

For conducting nanoparticles, nanocolloids, nanowires, and nanotubes, the quasistatic electric field associated with the SAW along the piezoelectric substrate can also play a role in their alignment and patterning along the substrate and in the fluid, complementing the acoustic radiation force acting on the particles that arises from the sound wave propagation through the fluid as the SAW leaks into it (see Section 2.1). In fact, it was shown that the nanotube assembly on the substrate surface was driven primarily due to the electric field, as a consequence of their polarizability under the standing SAW‐induced electric field. Under this field, the nanotubes tended to align along the electric field lines,^[^ ^119^, ^120^, ^121^ ^]^ with the possibility for further fine‐tuning the assembly afforded by rotating the nanotube bundles through adjustment of the SAW amplitude and phase.^[^ ^218^ ^]^ A similar alignment mechanism also exists in the bulk of the liquid due to dielectrophoretic forces acting on the nanotubes that arise under the non‐uniform evanescent electric field in the liquid phase emanating from the SAW along the substrate.^[^ ^172^, ^173^, ^219^ ^]^ Miansari et al., on the other hand, demonstrated the possibility for deagglomerating carbon nanotube bundles in air through a two‐step mechanism. The first involved the large mechanical impact arising from the $O(10^{8}$ m s^−2^) substrate surface acceleration, followed by coulombic fission under the electric field along the substrate due to its piezoelectricity, that dispersed them into 1‐µm dimension bundles, which were then aligned under mechanical shear.^[^ ^122^ ^]^

### 3.2 Exfoliation

**Figure 10:**
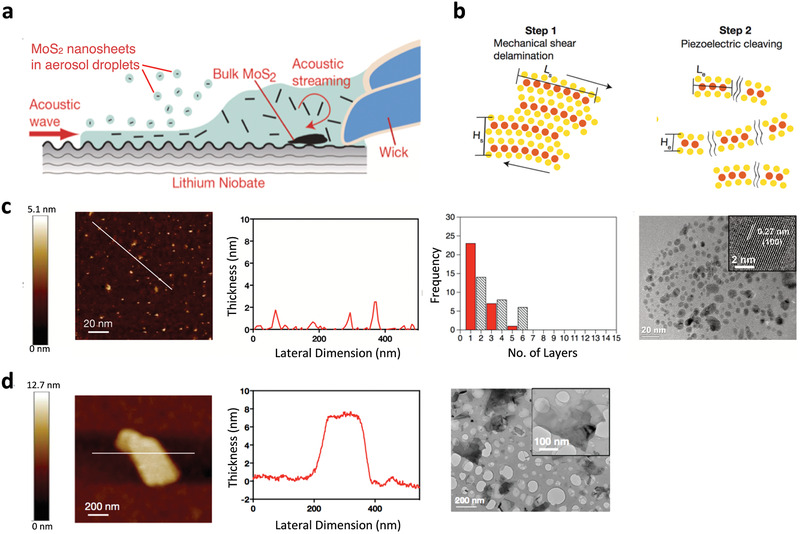
Exfoliation of 2D TMD nanosheets. a) Experimental setup showing the SAW or SRBW drawing a liquid suspension of bulk MoS_2_ powder through a wick onto the device where it forms a meniscus prior to being nebulized into micron‐dimension aerosol droplets containing the exfoliated MoS_2_, which are then collected for subsequent characterization. b) Two‐step mechanism by which the exfoliation is hypothesized to occur. The first step involves the mechanical delamination of the large powders into intermediate sheets with 100 nm order lateral dimensions *L_s_* and 10 nm thicknesses *H_s_* due to the large shear stress (on the order 10^4^ s^−1^) associated with the acoustic streaming within the liquid meniscus. The second step, which only arises if the material is piezoelectric (e.g., nanosheets with odd numbers of layers), involves the cleaving of the aforementioned intermediate sheets into thinner structures of 10 nm order lateral dimension *L*_*e*_ and 1 nm order thickness *H*_*e*_ (single‐ or few‐layers) due to the strong mechanical vibration acting at the end planes of sheets that is enhanced by the inherent electric field associated with electromechanical coupling of the acoustic waves in the nanosheets. c) Left to right: AFM image, lateral size distribution, frequency distribution (as a function of the number of layers), and transmission electron microscope image of the exfoliated MoS_2_ (piezoelectric when present in odd numbers of layers) nanosheets. d) Left to right: AFM image, lateral size distribution, and frequency distribution (as a function of the number of layers) of the exfoliated graphite (non‐piezoelectric) sheets. Reproduced with permission.^[^ ^123^ ^]^ Copyright 2018, Wiley‐VCH.

The role of the electric field, and, more broadly, the electromechanical coupling of the acoustic excitation, is particularly apparent when the material is itself piezoelectric. When bulk transition metal dichalcogenides (TMDs) such as molybdenum disulfide MoS_2_ or tungsten disulfide WS_2_, for example, were subjected to the SAW or SRBW nebulization (**Figure** **10**a), the material was shown to be efficiently exfoliated within a mere several seconds into single‐ and few‐layer nanosheets with lateral dimensions on the order of several tens of nanometers (Figure 10c).^[^ ^123^ ^]^ This is in stark contrast to exfoliation using sonication processes which typically require tens of minutes to many hours. More interestingly, even–odd asymmetry was observed in the number of layers possessed by the nanosheets that were exfoliated through a two‐step multiscale mechanism. The first step arises as a consequence of the large shear stress, on the order of 10^4^ s^−1^, generated by the acoustic streaming induced in the liquid meniscus as a result of the large $O(10^{8}$ m s^−2^) surface acceleration on the substrate (Figure 10a). This resulted in mechanical delamination of the 3D bulk crystals into intermediate sheets with 100 nm order lateral dimensions *L*_*s*_ and thicknesses *H*_*s*_ on the order of 10 nm (step 1 in Figure 10b). A second step then ensues in which these intermediate structures are subsequently cleaved into thinner and smaller single‐ and few‐layer nanosheets with 10 nm order lateral dimensions *L*_*e*_ and 1 nm order thicknesses *H*_*e*_ (step 2 in Figure 10b). The cleaving was attributed to the strong mechanical vibration acting at the end planes of the sheets, enhanced by the inherent electric field associated with odd‐layer nanosheets that are piezoelectric in nature due to the non‐centrosymmetric structure of TMDs.^[^ ^220^ ^]^ Fewer nanosheets with odd numbers of layers compared to nanosheets with even numbers of layers, in addition to an abundance of single‐layer nanosheets, were therefore produced. Such an assertion was supported by the observation that graphite—a centrosymmetric material—could only be delaminated into the intermediate sheets with dimensions *L*_*s*_ and *H*_*s*_ in the first mechanical delamination step (Figure 10d).

Further evidence of the role of the electric field and piezoelectricity in the exfoliation process was shown not only through density functional theory simulations but also experimentally by suppressing the electric field. By doing so, it could be seen that the predominantly monolayer nanosheets of 10 nm lateral dimension became progressively larger and thicker toward the 100 nm wide and 10 nm thick intermediate structures similar to that in Figure 10d with decreasing electric field intensity.^[^ ^123^ ^]^ While the evanescent electric field and microcentrifugation flow arising from the SAW (Figure 6) could also be employed to carry out the exfoliation,^[^ ^125^ ^]^ this was not as efficient a process in terms of the monolayer yield. The process also involved far greater processing times (around 30 mins), compared to a few seconds with the nebulization process. Moreover, it was shown that the monolayer yield in this process could be improved from around 2–3% up to approximately 10% by successively collecting the aerosols containing the exfoliated material and renebulizing them with up to four nebulization–condensation–renebulization cycles.^[^ ^124^ ^]^ Alternatively, the nebulization constitutes a rapid method for spray‐coat deposition of these 2D materials for fast simultaneous exfoliation and direct writing over large coverage areas on a variety of arbitrary surfaces.

**Figure 11:**
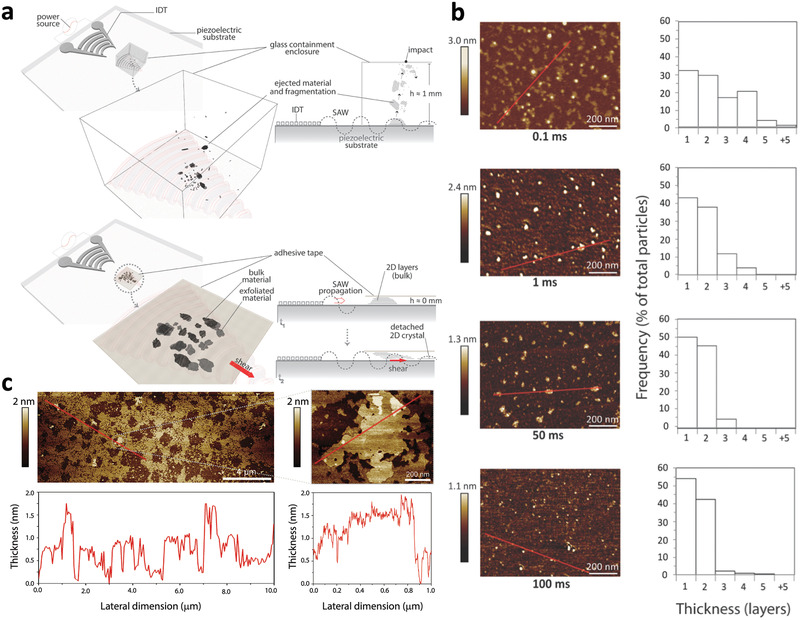
Dry exfoliation of TMD QDs and large flakes. a) Experimental setup: The top configuration, which facilitates the production of QDs, involves housing the bulk MoS_2_ powder feedstock in a chamber placed atop the SAW or SRBW device, whereas the bottom configuration, which facilitates the production of large micron‐dimension nanosheets, involves confining the feedstock under adhesive tape. b) Top to bottom: AFM images, lateral size distributions, and frequency distribution as a function of the number of layers of the QDs produced with the first configuration; successive rows correspond to results for different SAW or SRBW excitation times of 0.1, 1, 50, and 100 ms. c) AFM images of the large micron‐dimension flakes with high surface coverage, produced using the setup associated with the second configuration. Reproduced with permission.^[^ ^126^ ^]^ Copyright 2019, Royal Society of Chemistry.

Besides facilitating the synthesis of 10 nm order lateral dimension single‐ or few‐layer TMD nanosheets, exfoliation of the bulk TMD powders into smaller quantum dots (QDs) as well as considerably larger micron‐dimension flakes with the SAW or SRBW was also demonstrated by exploiting a different mechanism which did not necessitate liquid solvents or additives.^[^ ^126^ ^]^ This dry exfoliation process was carried out by confining the bulk feedstock powder within a miniature millimeter dimension chamber atop the SAW device for the production of MoS_2_ QDs, or, under adhesive tape (zero chamber height limit) for the production of the large micron‐dimension MoS_2_ flakes (**Figure** **11**a). The exfoliation mechanism in either case, nevertheless, did not involve the role of the electric field and hence piezoelectricity of the material as in the aforementioned case of nanosheet exfoliation via SAW or SRBW nebulization, but was rather governed by mechanical effects. Confinement of the bulk feedstock within the chamber allowed them to be successively ejected from the substrate surface under the surface acceleration associated with the SAW or SRBW, therefore, repeatedly impacting the walls of the enclosure before being re‐ejected from the surface. It was shown that the impact force during these cycles (approximately 100 nN) was typically on the order of that required to rupture the MoS_2_ flakes, therefore resulting in successive reduction in their dimension, both laterally as well as in thickness, thus facilitating efficient production of predominantly single‐ or two‐layer QDs. The longer the SAW or SRBW exposure time, and hence the greater the number of impact cycles, the thinner the QDs that were generated (Figure 11b). On the other hand, suppressing any ejection of the powders under the adhesive tape allowed the shearing of individual layers from the flakes as the SAW or SRBW traverses along the surface of the substrate. This then led to their progressive delamination into large but thin (mostly monolayer) pristine micron‐dimension flakes with very high (≈80%) surface coverage (Figure 11c).

The ability for flexible tuning of the lateral sheet dimensions into QDs or larger nanosheets and even large micron‐dimension flakes is an added advantage over conventional ultrasonic exfoliation methods, which either produce particles with very small lateral dimensions (i.e., QDs) in the case of probe sonication,^[^ ^221^, ^222^, ^223^, ^224^, ^225^ ^]^ or nanosheets with larger lateral dimensions but which possess several layers in thickness in the case of bath sonicators,^[^ ^226^, ^227^, ^228^, ^229^, ^230^ ^]^ the latter therefore necessitating the use of intercalation agents that can be challenging to remove post‐synthesis, and therefore compromising the purity and quality of the exfoliated product. In addition, the rapid process (seconds) also allows for comparable production quantities to that achievable with conventional sonication, which typically requires many hours.

### 3.3 Electronic Manipulation

In addition to the ability to exfoliate these nanomaterials, the SAW or SRBW has also been shown to facilitate reversible modulation of their electronic band structures without altering crystal structure or composition, therefore constituting a powerful tool for the tuning of electronic and optoelectronic devices. This continues from a long history on investigations on the SAW as an electromechanical wave, particularly in thin semiconductors (e.g., cadmium sufide) in the presence of a 2D electron gas close to its surface.^[^ ^127^ ^]^ The nonlinear interaction between the electric field associated with the SAW propagating along the surface of the piezoelectric material with the free charges in the semiconductor then gives rise to a current that is proportional to the acoustic intensity known as the acoustoelectric current.^[^ ^231^, ^232^, ^233^ ^]^ Subsequently, the Rayleigh SAW, viewed as a moving electrical superlattice potential, was shown to modify the electronic dispersion of ballistic graphene close to the charge‐neutrality point^[^ ^234^, ^235^, ^236^ ^]^ with an acoustoelectric current that is dependent on the SAW propagation direction.^[^ ^237^, ^238^, ^239^ ^]^ Similar acoustoelectric coupling has also been observed in graphene ranoribbons,^[^ ^240^, ^241^ ^]^ QDs,^[^ ^242^, ^243^, ^244^, ^245^ ^]^ suspended quantum point contacts,^[^ ^246^ ^]^ GaAs^[^ ^247^ ^]^ and GaAs/AlGaAs hetrostructures,^[^ ^248^, ^249^ ^]^ and, more recently, 2D materials such as MoS_2_^[^ ^250^ ^]^ and black phosphorous,^[^ ^251^ ^]^ in which an anomalous acoustoelectric current was sustained.

**Figure 12:**
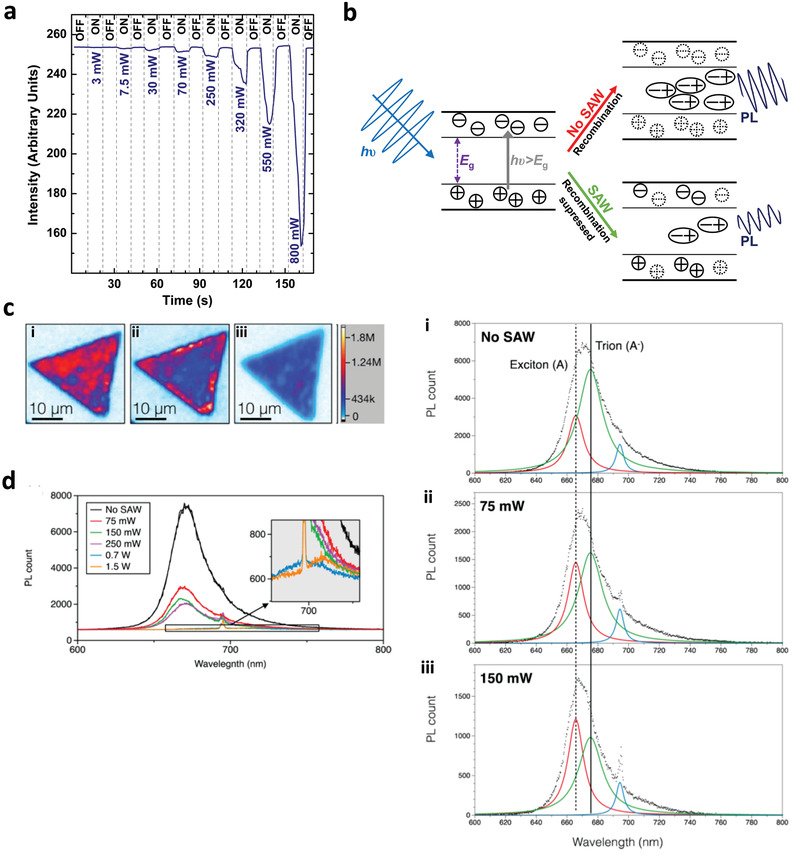
SAW or SRBW bandgap modulation in TMD nanosheets. a) Reversible PL modulation of a quasi‐2D MoS_2_ nanosheet under SAW or SRBW excitation. b) Mechanism by which electron recombination is suppressed by the SAW or SRBW. c) Images showing the PL response on a pristine monolayer MoS_2_ nanosheet, and d) corresponding PL spectra i) in the absence of SAW or SRBW excitation and in the presence of SAW or SRBW excitation at ii) low power (75 mW) and iii) high power (150 mW), respectively. a) Reproduced with permission.^[^ ^130^ ^]^ Copyright 2015, Wiley‐VCH. b,c,d) Reproduced with permission.^[^ ^131^ ^]^ Copyright 2016, American Chemical Society.

Remarkably, the SAW or SRBW was shown to influence both multilayer (quasi‐2D) and mono‐ or few‐layer (2D) sheets. In multilayered MoS_2_ samples, for example, a collective reversible reduction in the photoluminescence (PL) of the sample was observed to be rapidly quenched upon relaxation of the acoustic excitation (**Figure** **12**a) as a result of the efficient electroacoustic coupling preventing electron recombination (Figure 12b).^[^ ^130^ ^]^ For pristine monolayer MoS_2_, on the other hand, it was shown that an intricate acousto−excitonic coupling exists that allows the charge carriers (electron–hole pairs generated via optical excitation) to be spatially separated through their transport by the acoustic wave to the edge of the MoS_2_ nanosheets. As a result of their dissociation due to the moving electric field, they remain trapped within the potential maxima and minima of the conductance and valence bands, therefore suppressing their recombination and leading to quenching of the PL signal (Figure 12c).^[^ ^131^ ^]^ This was, however, conducted at room temperature in contrast to that previously shown for 2D electron gas systems based on GaAs layered structures at near zero Kelvin temperatures.^[^ ^128^, ^129^ ^]^ More interestingly, it was observed that this effect, similar to the acoustoelectric current, was also dependent on the applied acoustic intensity. At low intensities, at least for nanosheets which possess odd numbers of layers and hence inherently display piezoelectric properties as discussed above,^[^ ^220^ ^]^ PL‐induced trions are observed to be ionized into electrons, as observed by the shift in the trion to exciton peak in the PL spectra in Figure 12d(i–iii), in addition to strong quenching of the signal. In contrast, a red shift in the PL was instead observed at higher intensities, as seen in the inset of Figure 12d, which can be attributed to thermal effects that arise as the SAW or SRBW energy is converted to heat.^[^ ^131^ ^]^ That both bandgap modulation effects at low and high acoustic intensities were absent in MoS_2_ nanosheets with even numbers of layers then strongly points to the role of the piezoelectric coupling between the mechanical vibration associated with the acoustic wave and its corresponding electric field in the substrate. These couple to produce a corresponding strain and hence electric field in the nanosheet, which, in turn, allows the quasi‐particles within to be manipulated. Additionally, similar effects have also been reported for a heterojunction p‐n diode configuration comprising overlapping p‐type black phosphorus and n‐type MoS_2_ sheets, wherein a three‐order of magnitude enhancement in the photocurrent was observed.^[^ ^132^ ^]^

## 4 Polymeric and Biological Materials

### 4.1 Drug Delivery

In ultrasound‐driven drug delivery strategies,^[^ ^252^, ^253^ ^]^ pulsed ultrasonic excitation can, for example, are employed to enhance transdermal delivery^[^ ^254^ ^]^; guide, target, and/or trigger the release of encapsulated drugs from ultrasound‐responsive microbubbles, liposomes and nanoparticles^[^ ^255^, ^256^, ^257^, ^258^ ^]^; detach adherent cells from a surface without requiring trypsinization^[^ ^259^ ^]^; trigger mechanotransduction events^[^ ^260^ ^]^ or even rotate microorganisms.^[^ ^261^ ^]^ This is usually through cavitation or the microstreaming produced by the ultrasonic irradiation. In similar ways, high‐frequency acoustic excitation can also be employed for the manipulation of biological systems particularly for therapeutic applications, albeit via a distinctively different mechanism. Besides the absence of cavitational effects in high‐frequency systems, the differences in mechanism and application can primarily be attributed to the significantly shorter penetration depths at higher frequencies (see Equation (1)). As such, high‐frequency acoustics do not facilitate deep penetration through skin and tissue for in vivo applications that conventional ultrasonic therapeutics allow, but can rather be more useful for in vitro or ex vivo applications. For example, transmitting the low‐intensity vibration associated with the SAW or SRBW through a fluid coupling layer^[^ ^179^, ^262^ ^]^ into cells or organisms within a petri dish, agar plate, or cell culture chamber was not only observed to facilitate cell–cell interactions,^[^ ^133^ ^]^ activate mechanosensitive ion channels,^[^ ^134^, ^135^ ^]^ stimulate exosome production in the internal cell machinery,^[^ ^136^ ^]^ drive cell migration along acoustotactic gradients,^[^ ^137^ ^]^ promote tissue oxygenation for wound healing,^[^ ^138^ ^]^ or trigger neuronal stimulation in nematodes,^[^ ^139^, ^140^ ^]^ but also to enhance cellular uptake of nanoparticles, molecules, and nucleic acids by several‐fold, while retaining very high viabilities (>97%).^[^ ^142^ ^]^ Unlike cavitation‐induced pore formation in sonoporation processes, which can often lead to some irreversible cell damage and apoptosis (with cellular viabilities as low as 60% having being commonly reported),^[^ ^263^, ^264^, ^265^ ^]^ the high‐frequency SAW or SRBW excitation does not induce pore formation but rather temporarily disrupts the membrane lipid structure^[^ ^141^ ^]^ or cytoskeletal structure,^[^ ^266^ ^]^ thus increasing its permeability sufficiently to allow efficient transmembrane molecular transport.^[^ ^142^, ^267^ ^]^ We note that such rapid healing of the membrane leading to the retention of high cellular viabilities has also been reported following GHz frequency excitation, although in that case, nanopore formation in the cell membrane was claimed as a consequence of the larger localized acoustic pressures that can be generated at higher frequencies.^[^ ^268^ ^]^

In contrast to the phase transitions that occur in the lipid structure as a consequence of cavitational shock waves,^[^ ^27^ ^]^ the membrane permeabilization effect induced by the SAW or SRBW, is however transient. The lipid structure immediately returns to its original state when the excitation is relaxed, therefore preserving the viability of the cells (we however note that the SAW, or bulk waves on superstrates, at higher intensities has also been utilized for cell lysis,^[^ ^143^, ^144^, ^145^ ^]^ particularly for the extraction of extracellular vesicles and exosomes). Moreover, as the internalization mechanism does not involve endocytosis, the therapeutic cargo was observed to be distributed throughout the cytosol instead of being localized within the endosomes/lysosomes, thus minimizing the possibility for their degradation through the endosomal recycling pathway and thereby facilitating higher probabilities for trafficking them to the nucleus where they can be transfected. Indeed, with siRNA delivery, two‐fold knockdown was observed in the gene expression,^[^ ^142^ ^]^ therefore highlighting the potential of the platform as a powerful tool for ex vivo autologous therapeutics in which a patient's target cells are isolated from their blood or tissue, re‐engineered in the laboratory and re‐infused to the same patient.

In a similar manner, direct application of the SAW or SRBW to tissue was shown to enhance controlled delivery of these therapeutic molecules into the mucosal layer.^[^ ^146^ ^]^ In contrast to bulk ultrasound transdermal delivery,^[^ ^254^ ^]^ such localization within the mucosa, which is rich in immunocytes, avoids deeper penetration into the vascularized submucosal regions where the drug is mostly taken up by the systemic circulation. As such, the technology can particularly be useful as a vaccination strategy, since a far stronger local immune response can be elicited compared to systemic vaccination routes. This was validated in a porcine buccal model in which the platform was demonstrated as a practical portable hand‐held applicator for effective non‐invasive vaccination.

### 4.2 Generation of Multicellular Bodies

Besides providing a means for enhancing flow perfusion and hence cell culture, the ability of the SAW‐induced streaming,^[^ ^147^ ^]^ and, in particular, microcentrifugation flow discussed previously in Section 2.2.1 can also be exploited to selectively concentrate (or, conversely, disperse) cells in a suspension (Figure 6c). This effect was employed for the assembly of embryoid bodies leading to the formation of cell spheroids (Figure 6d)—3D spherical cell aggregates displaying cell–cell and cell–matrix interactions, which more closely mimic in vivo avascular tumor structure and functionality—whose size can be tuned through the input SAW power and hence the flow intensity.^[^ ^148^ ^]^ We note too the possibility for generating similar cellular aggregates was later demonstrated by trapping the cells within the nodes of standing SAWs; it is nevertheless considerably harder to obtain size control in this case without changing the SAW wavelength, which necessitates separate devices with different frequencies or devices with variable frequency through the use of tapered interdigitated transducers, although excitation of different frequencies in this manner occurs at different spatial locations on the chip.^[^ ^149^, ^150^ ^]^

### 4.3 Nanoparticle Synthesis and Encapsulation

**Figure 13:**
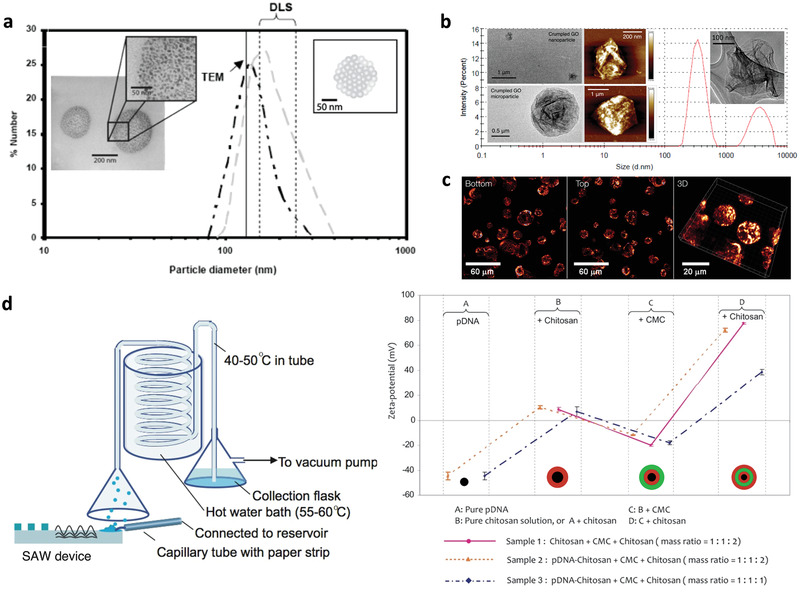
Particle synthesis via SAW or SRBW nebulization. The top row reports particle size distributions that confirm production of a) 100 nm order polymer nanoparticles comprising a cluster of sub‐50‐nm aggregates (see insets), and b) crumpled GO nanoparticles and microparticles, simply by nebulizing solutions comprising the polymer or GO suspension, respectively. Also shown in (c) is evidence through confocal microscopy sectioning of the possibility of simultaneously encapsulating, in this case, a fluorescent protein, within the polymer particles. d) The bottom row shows a schematic of the experimental setup (left) for the synthesis of multilayer polymer nanocapsules by nebulizing a polymer solution (in this case, chitosan), collecting the dried particles in a complementary polymer solution of opposite charge (in this case, carboxymethylcellulose [CMC]),and renebulizing over many cycles (one cycle per layer); each layer as well as the plasmid DNA (pDNA) encapsulated within them was verified from the alternating‐zeta potentials measured after the formation of each layer (right). a,b) Reproduced with permission.^[^ ^151^ ^]^ Copyright 2008, IOP Publishing Ltd; c) Reproduced with permission.^[^ ^153^ ^]^ Copyright 2009, American Institute of Physics; d) Reproduced with permission.^[^ ^155^ ^]^ Copyright 2011, American Chemical Society.

The generation of micron‐dimension aerosols comprising a polymer or biological solution using the SAW or SRBW, following which the solvent evaporates in‐flight to leave behind 100 nm dimension polymer or protein nanoparticles, is a convenient means for facile and rapid nanoparticle production akin to spray drying synthesis but without requiring energy‐intensive heating as a drying step^[^ ^151^, ^152^, ^153^, ^154^ ^]^ (**Figure** **13**a). Parenthetically, we note that the evaporation of the aerosol droplets also provides a useful means for concurrently crumpling graphene oxide (GO) nano‐ or micro‐sheets (Figure 13b) simply by nebulizing a GO suspension.

Without any adaptation of the setup, the nebulization technique further lends itself to the possibility for simultaneously encapsulating drug molecules within the polymeric particles during their synthesis as a one‐step production method simply by suspending the therapeutic moieties within the polymer solution (Figure 13c). Multiple layers of polymer encapsulants, each of which can be judiciously chosen to controllably tune the drug release profile, can also be easily coated around the drug without requiring sacrificial templating or lengthy and cumbersome layer‐by‐layer deposition.^[^ ^269^ ^]^ This was carried out by collecting the condensed nebulized polymer nanoparticles in a complementary polymer solution of alternate charge and renebulizing them (Figure 13d), each successive nebulization–condensation cycle therefore adding an additional polymer layer over the previous layer, although we note that the complexation between additional polymer layers actually leads to slightly smaller nanocapsule sizes.^[^ ^155^ ^]^

### 4.4 Patterning and Deposition

**Figure 14:**
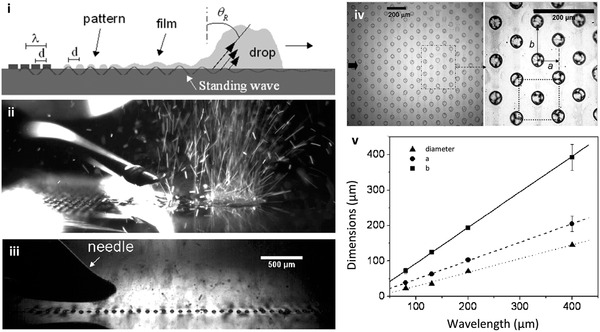
SAW/SRBW surface patterning. i) Schematic depiction and ii) snapshot of the nebulization of a polymer solution from a trailing film of a translating drop (from left to right in the image) dispensed from a needle onto the piezoelectric substrate (both of which occur due to the travelling wave component of the acoustic wave) to leave behind polymer spots on the substrate that are arranged in a regular 2D hexagonal closed pattern (iii, iv), whose v) dimension as well as lateral spacing *a* and *b* highly correlates with the wavelength of the SAW/SRBW, as set by the IDT spacing *d*, and hence frequency. Reproduced with permission.^[^ ^158^ ^]^ Copyright 2008, American Chemical Society.

Beyond concurrent synthesis and encapsulation, it is possible to exploit the SAW and SRBW devices as a means for simultaneous in situ administration to facilitate direct inhalation for pulmonary drug uptake,^[^ ^164^ ^]^ or as a spray deposition technique, among other applications. The latter, for instance, includes therapeutic delivery where drugs could be applied via spraying onto skin or mucosal surfaces, industrial applications such as thin spray coating of functional thin films,^[^ ^156^ ^]^ or biological assays such as protein spot patterning onto microarrays.^[^ ^157^ ^]^ Moreover, it is possible to extrude thin films of polymer solutions using the SAW or SRBW, whose simultaneous nebulization under the acoustic excitation leaves behind highly regular polymer spot patterns on the substrate whose spatial distribution and setup is closely correlated and hence easily tuned with the acoustic wavelength and thus the applied frequency (**Figure** **14**).^[^ ^158^ ^]^ Similar patterns can also be achieved by imposing standing waves on the polymer film or hydrogel through the excitation of an underlying standing SAW.^[^ ^159^, ^160^ ^]^

**Figure 15:**
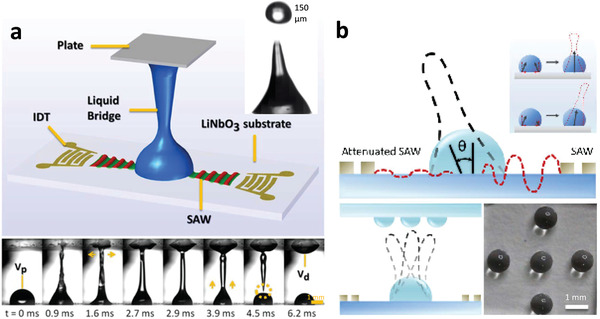
Dispensing and patterning enabled by SAW or SRBW jetting. a) As illustrated by the schematic (top) and sequence of images (bottom), the jetting of a parent drop can be employed for the ejection and hence dispensing of drops (see inset), or if impeded by a top cover plate, the formation of a capillary bridge, whose spreading onto the top plate (depicted by the horizontal arrows), and subsequent retraction and pinch‐off (dotted circle) leaves behind a spot on the top plate whose volume *V*_*d*_ is closely related to the parent drop volume *V*_*p*_. b) The angle of the jet *θ* can be controlled by an asymmetric imbalance of the input power to both IDTs; the jet is vertical (*θ* = 0) when equal power is delivered to both IDTs, or biased toward the IDT with the lower power (top). By repeatedly pulsing the signal and altering the relative power to both IDTs sequentially, it is then possible to jet and hence dispense drops at different locations on the top plate to create a regular array pattern (bottom left and right). Reproduced with permission.^[^ ^162^ ^]^ Copyright 2019, American Chemical Society.

Alternatively, if patterning on the piezoelectric substrate is undesirable, it is also possible to jet single droplets (**Figure** **15**a) from a drop reservoir toward the desired spotting plate,^[^ ^161^ ^]^ or from a microchannel for injection into a sample characterization chamber.^[^ ^163^ ^]^ Moreover, the jetting technology is amenable for dispensing cells down to single cell resolution. The technology then constitutes a means for 3D bioprinting,^[^ ^161^ ^]^ for example, or to form capillary bridges with a top plate^[^ ^270^ ^]^ that subsequently pinches‐off to leave behind the deposited spots (Figure 15b). Given that the angle of the jet can be adjusted through asymmetric delivery of the input signal into the parent drop reservoir, it is possible to dispense and hence produce multiple spots at different locations without moving the print head (i.e., the SAW or SRBW chip).^[^ ^162^ ^]^

As cavitation is not responsible for the nebulization (or jetting, for that matter) at the high frequencies employed,^[^ ^209^, ^271^ ^]^ the large GPa pressures and temperatures generated during collapse of the cavitation bubbles, which constitute the mechanism for aerosol generation in conventional ultrasonic nebulization,^[^ ^35^ ^]^ and which could lead to considerable damage particularly to large molecules such as biologics (e.g., DNA, RNAi, peptides, proteins) or stem cells, are typically absent.^[^ ^79^, ^164^, ^165^, ^166^, ^167^, ^168^ ^]^ Moreover, the time periods over which the applied field reverses, which scale as the inverse of the SAW or SRBW frequency, are typically shorter than the hydrodynamic molecular relaxation time scales such that hydrodynamic shear effects on molecular unfolding and hence denaturation are negligible.

## 5 Summary

In the same way that sonochemistry driven by bulk kHz‐order ultrasound has opened up a myriad of possibilities for the processing of materials ranging from crystallization, polymerization and gelation to the production of nanostructured materials in the past three decades, the recent studies reported in this paper that reveal the use of high‐frequency (MHz‐order) surface and hybrid acoustic wave excitation to manipulate and synthesize 2D and bulk crystals, as well as various polymeric and biological materials, offer a glimpse of an emerging field of phonon‐mediated chemical and biochemical synthesis. As with all microfluidic devices and micro‐electro‐mechanical‐systems,^[^ ^272^, ^273^ ^]^ such miniaturized chip‐scale technology is typically limited by the small volumes that can be processed, although scaling the platform for high‐throughput commensurate with industrial‐scale production rates is afforded through process intensification techniques involving massive parallelization of the acoustic wave chips. This, in particular, is made possible by exploiting the economies of scale associated with mass nanofabrication through which devices can be fabricated at costs down to typically US$1 each. Moreover, given their energy efficiency, enhanced yield, high selectivity, and, in some cases, capacity to minimize or altogether remove the requirement of harsh solvents or etchants from the process, we expect these methods to herald greener and more sustainable processing techniques.^[^ ^274^ ^]^

Owing to the higher applied frequencies and the significantly lower powers employed, cavitation is largely absent and hence the dominant mechanism responsible for these materials synthesis and manipulation processes is fundamentally distinct to that for cavitation‐driven sonochemical methods. Besides the possibility of retaining high macromolecular and cellular viability, which constitutes a significant advantage over conventional ultrasound‐driven technologies in biological processes, this is anticipated to lead to further discoveries of new processing methods and unique material phases and structures. While the evanescent electric field emanating from the piezoelectric substrate, together with the unprecedentedly large 10^8^ m s^−2^ order acceleration corresponding with its surface displacement, feature repeatedly and dominantly in many of the observed phenomena, the precise manner by which this nonlinear electromechanical coupling arising from the phonon source transfers energy across multiple length or frequency scales has yet to to be explicated. In particular, how the large 100 µm order wavelengths associated with the MHz‐order excitation allow manipulation, assembly, polarization, and ordering at sub‐nm‐order molecular length scales or THz‐order molecular vibration frequencies remains unexplained. Further work to elucidate the governing mechanisms that underpin such nonlinear spectral energy cascading will therefore facilitate not just a better fundamental understanding of these remarkable phenomena, but will also be instructive in how these systems can be optimized to obtain further gains in yield and efficiency.

## Conflict of Interest

The authors declare no conflict of interest.
